# ZCAT: Zero-shot cross-crop annotation transfer–A new paradigm leveraging plant organ similarity

**DOI:** 10.1016/j.plaphe.2026.100267

**Published:** 2026-08-13

**Authors:** Yuxiang Zeng, Tingting Chen, Weimeng Fu, Wenting Wang, Song Chen, Guanfu Fu

**Affiliations:** State Key Laboratory of Rice Biology and Breeding, China National Rice Research Institute, Hangzhou, 310006, China

**Keywords:** Semantic segmentation, Pseudo-mask screening, Domain adaptation, Wheat spike segmentation, Human-in-the-loop learning

## Abstract

The inflorescence is a key yield-determining organ, yet its complex morphology makes manual pixel-level annotation time-consuming, leading to a scarcity of high-quality segmentation datasets. To address this bottleneck, we propose ZCAT (Zero-shot Cross-crop Annotation Transfer), a novel paradigm for zero-annotation cross-crop pseudo-mask screening. ZCAT completely eliminates pixel-level manual annotation of the target crop, requiring only holistic quality assessment of model-generated pseudo-masks (5–10 s per image). Specifically, we train a SegFormer model on public rice panicle datasets (CVRP and RiceSEG) and transfer it across crops to the wheat spike segmentation task. The key innovation is the introduction of a human-defined quality function Q, which circumvents the fundamental challenge in self-learning algorithms: the inability of computers to autonomously distinguish good masks from bad ones. Through iterative human-in-the-loop pseudo-label screening with a curriculum learning strategy, each round adds only a few high-quality pseudo-masks to the training set, continuously improving model performance. After four iterations, ZCAT produces pseudo-masks with an average Spike IoU of 0.7003, evaluated against the GWFSS manual annotations as ground truth. Moreover, the pseudo-mask dataset exhibited higher benchmark performance than the GWFSS manual annotations (Spike IoU 0.7612 vs. 0.7027; mIoU 0.8627 vs. 0.8247), suggesting stronger self-consistency. A generalization test on a strictly held-out set of 100 manually annotated wheat spike images showed that the model trained on ZCAT-generated pseudo-masks achieved marginally better performance than that trained on the GWFSS manual annotations (Spike IoU: 0.5112 vs. 0.4927; mIoU: 0.5627 vs. 0.5247). The time budget of the ZCAT pipeline was substantially lower than that of manual annotation. ZCAT opens a new pathway for rapid annotation of plant reproductive structures or other organs and significantly reduces data preparation costs in plant phenomics. The generated wheat spike pseudo-mask dataset and the mask quality screening tool (Mask Quality Screener) are open-sourced.

## 1 Introduction

Accurate image-based recognition of crop yield organs is critical for agriculture, making semantic segmentation of reproductive organs a current hotspot. However, public pixel-level datasets for plant reproductive organs remain extremely scarce. Zhou et al. [1] reported that pixel-level annotation of rice panicles takes 0.5–1.5 h per image, with total annotation time exceeding 3000 h for the whole dataset. Tang et al. [2] noted that the lack of high-quality panicle annotation datasets severely hinders the application of advanced computer vision algorithms in plant phenomics. Such high labor costs limit the availability of organ-level segmentation datasets, creating an urgent need for zero-annotation or weakly supervised methods. Currently, only three open-source datasets provide pixel-level semantic annotations for rice panicles (CVRP [2], RiceSEG [1], and the Paddy rice imagery dataset [3]), while for wheat spikes only GWFSS [4] provides pixel-level annotations. Even large-scale datasets such as the FIP 1.0 [5] lack pixel-wise semantic masks.

Researchers have explored alternative methods such as synthetic data and pseudo-labels to accelerate annotation and reduce labor costs. Barth et al. [6] showed that synthetic images from 3D modelling can automatically provide pixel-level annotations, enabling large-scale training data expansion. Magistri et al. [7] pointed out that domain drift between training and test environments degrades performance, and re-annotating data for each new field is impractical. Luo et al. [8] identified limited annotated samples as a major obstacle for deep learning in agriculture. This challenge is echoed in a recent survey by Meraj et al. [9]. Asada et al. [10] noted that even state-of-the-art zero-shot models like SAM perform poorly on leaf segmentation due to dense repetitive textures. They achieved training data without manual annotation using L-systems and GANs, but still required predefined rules.

We observe that both manual annotation and model-inferred pseudo-labels ultimately require human verification. Cao et al. [11] stated that “label quality sometimes outweighs label quantity”, completing quality screening of pseudo-masks for thousands of images in just 6 h. Tang et al. [2] adopted a “model prediction + manual correction” workflow. Inspired by this, we propose that if pseudo-labels can produce high-quality semantic annotations, simply selecting high-quality pseudo-masks manually can rapidly generate new datasets, saving valuable annotation time.

Plant organs share similarities–leaves, flowers, and fruits across different crops have common characteristics. This inspires us to leverage public annotated datasets of one crop to perform pseudo-label inference on unannotated new crops, thereby rapidly generating organ-level datasets, an idea inspired by Zhang et al. [12], who successfully used GANs to transfer fruit annotations across species in their EasyDAM method. However, as emphasised by Tang et al. [2], even within the same crop (rice), panicle segmentation faces substantial challenges due to illumination, occlusion, weed interference, and inter-variety differences. Transferring a panicle segmentation model from rice to wheat, two crops with significantly different organ morphologies, is even more challenging and requires a new paradigm. This study addresses this challenge by proposing ZCAT.

Currently, only one manually annotated wheat spike semantic segmentation dataset exists (GWFSS [4]). We train wheat spike segmentation models using existing rice panicle annotation datasets (CVRP and RiceSEG), perform pseudo-mask inference on wheat spikes, and compare the pseudo-masks with GWFSS manual annotations, thereby simulating a method to rapidly generate a dataset comparable to manual annotations in the absence of a wheat spike semantic annotation dataset.

The main contributions of this study are:

(1) ZCAT enables cross-crop transfer without pixel-level annotations on the target domain, leveraging plant morphological similarity between source and target organs, unlike existing methods that require generative models [12], synthetic data [6,10], or predefined rules [10].
(2) We introduce a human-defined quality function Q that solves the fundamental bottleneck in self-learning: the inability of computers to autonomously distinguish good pseudo-masks from bad ones, a challenge that cumulative pseudo-label refinement approaches [11] fail to address.
(3) We demonstrate that human-guided non-accumulating pseudo-mask screening can produce a wheat spike segmentation dataset with generalization ability test slightly higher than manual annotations, while reducing annotation time from minutes to seconds per image.

## 2 Materials and Methods

### 2.1 Datasets

**Fig. 1:**
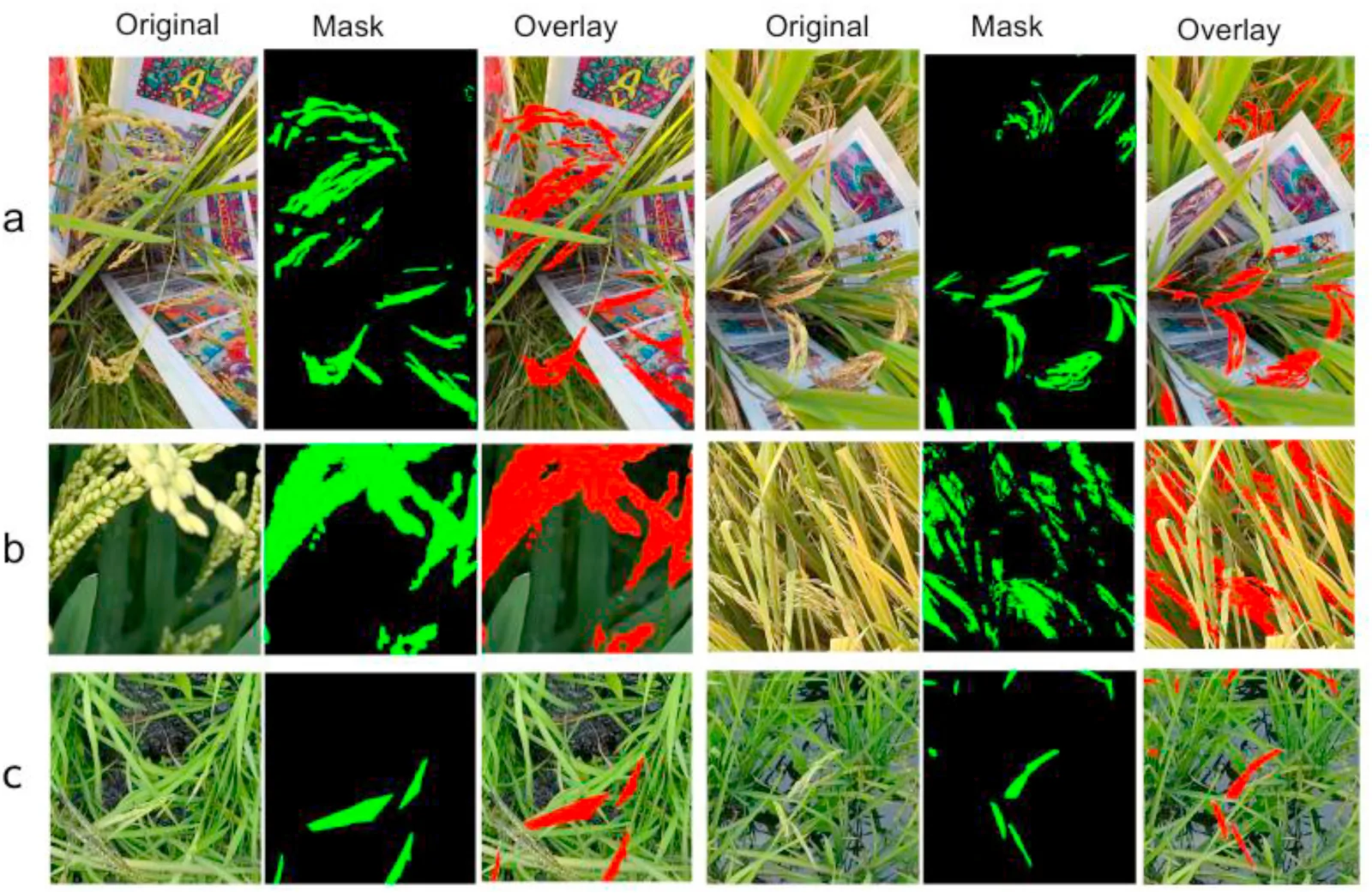
Example images from three open-source rice panicle annotation datasets used in this study: (a) CVRP dataset; (b) RiceSEG dataset; (c) Paddy rice imagery dataset.

We used four open-source datasets: three rice panicle semantic segmentation datasets (CVRP, RiceSEG, Paddy rice imagery) and one wheat spike semantic segmentation dataset (GWFSS) (Table 1). All images are RGB with varying resolutions, annotated with pixel-level panicle/spike masks. CVRP [2] contains 2292 rice panicle images from 231 landraces and 50 modern cultivars. RiceSEG [4] contains 565 images from 5 countries covering >6000 genotypes. The Paddy rice dataset [3] contains 5865 UAV images; some are blurry with incomplete annotations (Fig. 1), so it is used only in partial experiments. GWFSS [4] is the only pixel-level wheat spike dataset, containing 739 images from 67 experimental sites across 11 countries, used as ground truth for evaluation. We aim to explore a new paradigm that, without using any ground truth wheat spike masks, can generate pseudo-labels that are comparable to human annotations.

**Table 1:** Summary of datasets used in this study.

Dataset	Crop type	Number of images with pixel-level panicle labels	Average panicle proportion per image	Panicle proportion range per image	Reference
CVRP	Rice (human label)	2292	7.42%	0.31% – 36.89%	[2]
RiceSEG	Rice (human label)	565	18.41%	0.00% – 69.90%	[1]
Paddy rice imagery	Rice (no human check)	5865	2.24%	0.41% – 13.89%	[3]
GWFSS	Wheat (human label)	739	14.49%	4.00 × 10^−4^% – 58.93%	[4]

### 2.2 Model architecture

We use SegFormer [13], a lightweight Transformer-based segmentation network. Its hierarchical Mix Transformer (MiT) encoder extracts multi-scale features, and its MLP decoder avoids complex dilated convolutions. Compared to CNNs, SegFormer achieves higher mIoU on plant organ segmentation tasks [1,2]. The self-attention mechanism helps capture long-range dependencies, facilitating recognition of cross-crop morphological similarities. The model uses ImageNet-pre-trained MiT-B0 as backbone, AdamW optimizer, and cross-entropy loss.

In this study, we train the SegFormer model on the combined rice panicle datasets (CVRP [2] and RiceSEG [1], totaling 2857 images) to perform spike segmentation. The training is conducted for 100 epochs with a batch size of 8, an initial learning rate of 6 × 10^−5^, and a weight decay of 1 × 10^−4^. Input images are resized to 512×512 pixels, and data augmentation includes random horizontal/vertical flips, rotations, affine transformations, and brightness/contrast adjustments. The best model is selected based on the validation foreground (spike) IoU, employing an early stopping criterion with a patience of 10 epochs. After training, the model is used to infer pseudo-masks on the GWFSS wheat spike dataset [4] and the GWHD2021 detection dataset [14], generating pixel-level spike annotations for these target-domain images without any manual pixel labeling on wheat.

### 2.3 ZCAT methodology

**Fig. 2:**
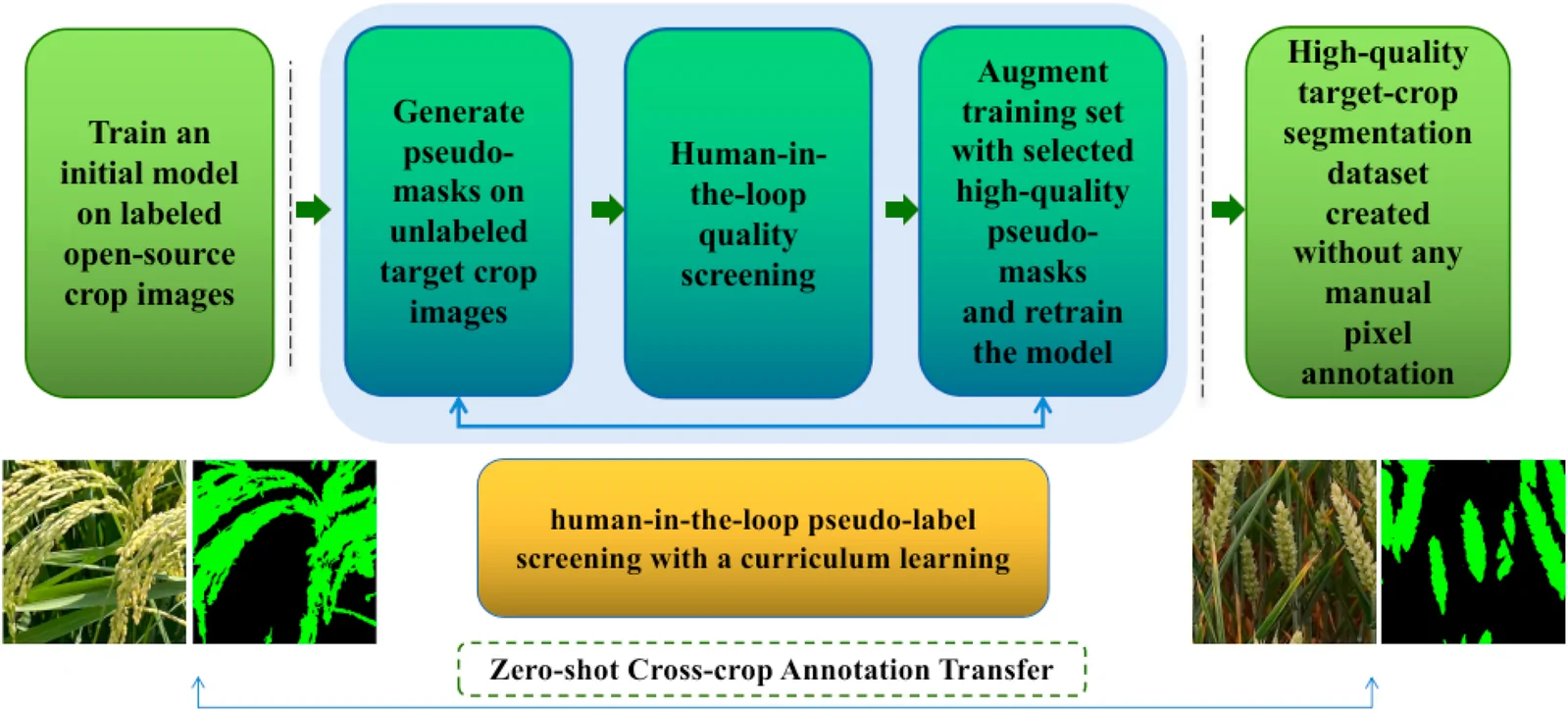
Workflow of ZCAT. Refer to the Materials and Methods section for the specific algorithm and formulas of ZCAT.

ZCAT is a cross-crop transfer framework that eliminates pixel-level manual annotation on the target crop. It can be applied to arbitrary source–target crop pairs. Here, we demonstrate it using rice as the source crop (rice panicle datasets) and wheat as the target crop (wheat spikes): an initial model is trained on the source, pseudo-masks are generated for the target, and the model is iteratively refined by adding only a small set of human-verified high-quality pseudo-masks to the training set. No pixel-level correction is ever performed on target images (Fig. 2).

#### 2.3.1 Initial model training (model A)

We merged the two open-source rice panicle datasets, CVRP [2] and RiceSEG [1], into a source domain set S_0_ containing 2857 images with pixel-wise semantic annotations of panicles. S_0_ serves as the “seed data”, providing the knowledge source for cross-crop transfer. Each sample in S_0_ is a pair (x_i_, y_i_), where x_i_ is an image and y_i_ is the corresponding manually annotated binary panicle mask. The total number of samples is denoted by N_0_ = 2857 (2292 from CVRP and 565 from RiceSEG).

$$S_{0}=\left\{\left(x_{i},y_{i}\right)\right\}\begin{array}{c} N_{0}\\ i=1 \end{array},{\;N}_{0}=2857$$

Using S_0_, we trained the initial SegFormer [13] model parameters θ_0_ (Model A). Here, θ includes all trainable parameters of the SegFormer architecture, such as the weights of the Transformer encoder and the parameters of the MLP decoder. In the training objective, L_CE_ denotes the cross-entropy loss function, and f_θ_(x) is the prediction output of the model (with parameters θ) for an input image x. The ground-truth label corresponding to x is y. Adjust the model parameters θ so that the sum of the cross-entropy losses between the model predictions fθ(x) and the ground-truth labels y is minimized. The set of parameters that achieves this minimum is θ_0_.

$$\theta_{0}=\underset{\theta }{\arg\;\min}\sum_{\left(x,y\right)\in S_{0}}L_{CE}\left(f_{\theta }\left(x\right),y\right)$$

#### 2.3.2 Pseudo-mask generation and human screening (round 1)

$$T={\left\{x_{j}^{\prime }\right\}}_{j=1}^{739}$$

T denotes the target-domain GWFSS wheat image set [4], which contains 739 images. ${\;x}_{j}^{\prime }$ represents the j-th image in the GWFSS dataset.

$${\hat{y}}_{j}^{\left(t\right)}=f_{\theta_{t-1}}(x_{j}^{\prime })$$

${\hat{y}}_{j}^{\left(t\right)}$ denotes the pseudo-mask predicted by the model for image $x_{j}^{\prime }$ in the t-th iteration round. $f_{\theta_{t-1}}(x_{j}^{\prime })$ means using the model from the previous round to infer a pseudo-mask for the target-domain image $x_{j}^{\prime }$.

$$Pt=\left\{\left(x_{j}^{\prime },{\hat{y}}_{j}^{\left(t\right)}\right)|{\hat{y}}_{j}^{\left(t\right)}{=f}_{\theta_{t-1}}\left(x_{j}^{\prime }\right),x_{j}^{\prime }\in T\right\}$$

Pt denotes the complete set of pseudo-masks generated in round t (including both good and bad quality masks).

$$H_{t}=\left\{\left(x_{j}^{\prime },{\hat{y}}_{j}^{\left(t\right)}\right)\in P_{t}|Q\left(x_{j}^{\prime },{\hat{y}}_{j}^{\left(t\right)}\right)=1\right\}$$

Ht represents the set of high-quality pseudo-masks selected in round t.

$$Q\left(x_{j}^{\prime },{\hat{y}}_{j}^{\left(t\right)}\right)=\left\{ \begin{array}{c} \;1\;\left(\text{good}\right)\\ 0\;\left(\text{bad}\right) \end{array}\right.$$

$Q\left(x_{j}^{\prime },{\hat{y}}_{j}^{\left(t\right)}\right)$ is the human quality screening function, which outputs 1 for a good mask and 0 for a bad mask. We apply Model A to all 739 wheat images in GWFSS ($T=\{{x_{j}^{\prime }\}}_{j=1}^{739}$) to get pseudo-masks ${\hat{y}}_{j}^{\left(1\right)}=f_{\theta_{0}}\left(x_{j}^{\prime }\right)$, forming P1 = {$\left(x_{j}^{\prime },{\hat{y}}_{j}^{\left(1\right)}\right)$}. Two researchers independently assess each pseudo-mask holistically (5–10 s per image, no pixel-level correction), judging it as “good” or “bad” based on boundary fit and obvious errors (consensus required). This yields $H_{1}=\left\{\left(x_{j}^{\prime },{\hat{y}}_{j}^{\left(1\right)}\right)\in P_{1}|Q\left(x_{j}^{\prime },{\hat{y}}_{j}^{\left(1\right)}\right)=1\right\}$, where Q is the human screening function. H1 (called good_set1) contains 157 high-quality images.

**Fig. 3:**
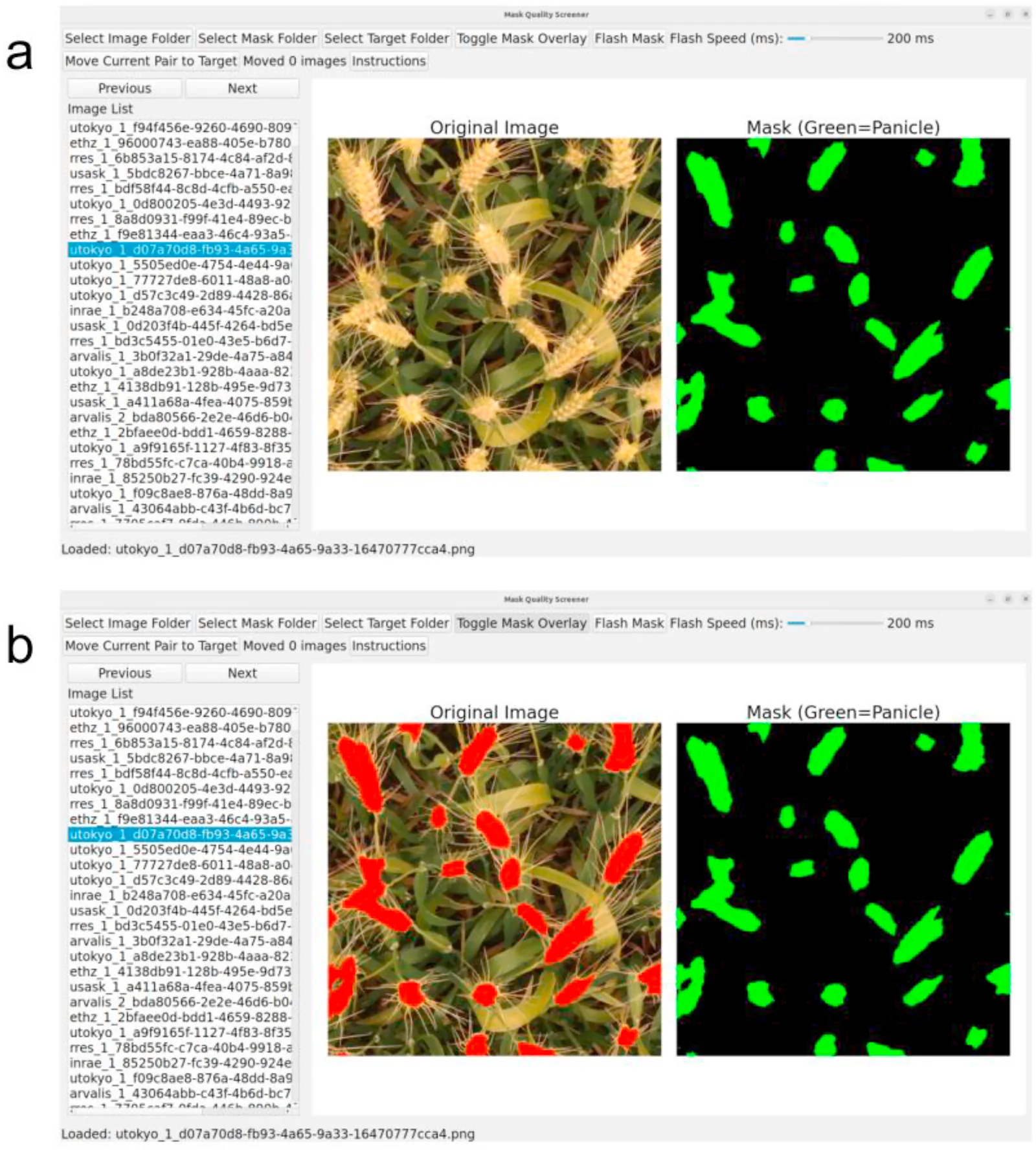
A mask quality screening tool, named “Mask Quality Screener”, was developed for easy spike mask quality evaluation. (a) The original image and spike mask are displayed simultaneously on the software UI. (b) The spike mask is overlaid on the original image to facilitate easy evaluation of the spike overlap with the original image. The spike mask can also be flashed on the original image to check the overlap (Supplementary File 1).

The Q function is a human-based visual inspection method that tells the computer which pseudo-masks are of high quality and which are not. It can be defined by one or multiple experts through holistic visual assessment of the pseudo-mask overlay on the original image. In this study, two researchers independently assessed each pseudo-mask using the “Mask Quality Screener” tool (Fig. 3), which allowed them to visually compare the pseudo-mask overlay on the original image and quickly evaluate boundary fit, false positives, and false negatives. Only the pseudo-masks that were independently labelled as “good” by both experts were retained to form the goodset and define the Q function for each iteration; inconsistent assessments were excluded to ensure quality control. This procedure was applied consistently across all screening rounds. Due to its subjective nature, different experts may select different numbers of pseudo-masks as the goodset (e.g., selection ratios ranging from ∼15% to ∼35% were observed in our experiments). However, the validity of Q is not determined by the absolute selection ratio; rather, it is verified by statistical comparison: in each iteration, the selected goodset must exhibit significantly higher Spike IoU than the unselected badset (p < 0.05, two-sample *t*-test). As shown in Supplementary Table 1–4, this condition was consistently satisfied across all four iterations of our ZCAT pipeline. Through multiple iterations, each round allows the model to learn from increasingly better pseudo-masks, thereby progressively improving the spike recognition accuracy.

#### 2.3.3 Iterative human-guided refinement (rounds 2–4)

For each iteration round t = 1,2,3,4 we do not accumulate previous high-quality sets. Instead, the training set for round t is always the original source data plus the newly screened set Ht:

$$S_{t}=S_{0}\cup H_{t}$$

S_t_ denotes the new training set after the t-th iteration round, and S_0_ represents the “seed data” (CVRP + RiceSEG). H_t_ denotes the set of high-quality pseudo-masks selected in the t-th round.

$$\theta_{t}=\underset{\;\theta }{\arg\;\min}\sum_{\left(x,y\right)\in S_{t}}L_{CE}\left(f_{\theta }\left(x\right),y\right)$$

$\theta_{t}$ represents the model parameters obtained after the t-th round of training. The formula indicates that in round t, the model is trained using the current training set St, and the model parameters θ_t_ for that round are obtained by minimising the loss. The new model f_θt_ is then applied to T to generate a fresh pseudo-mask set P_t+1_, from which we screen a new high-quality set H_t+1_ using the same Q function.

This process embodies curriculum learning: the model first learns from the “easiest” wheat spikes (those most similar to rice panicles), and as its capability improves, we feed it more challenging examples from the next round. The human screening provides an oracle that judges the correctness of pseudo-masks, guiding the model training.

Following this strategy, we obtained: H_2_ (good_set2) with 179 images, used for Model C. H_3_ (good_set3) with 242 images, used for Model D. H4 (good_set4) with 230 images, used for Model E. Thus: Model B trained on S0 ∪ H1, Model C trained on S0∪ H2, Model D trained on S0∪ H3, Model E trained on S0∪ H4.

To validate the effectiveness of the Q function in each iteration, we performed statistical analyses comparing the Spike IoU and mIoU between the goodset and badset using a two-sample *t*-test. This statistical verification was applied to ensure that the human visual screening approach consistently selected a goodset of superior quality to the badset. In practice, if the Spike IoU difference between the goodset and badset becomes negligible, we considered the selection criteria too lenient and adopted a stricter screening strategy to extract pseudo-masks with more true positives and fewer false positives/false negatives. The detailed statistical results for each iteration are provided in Supplementary Table 1–4.

The rationale for the non-accumulating iteration design (St = S0 ∪ Ht) is grounded in the observed pseudo-mask quality hierarchy (H4>H3>H2>H1). Critically, the performance of S0∪ H4 surpassed that of the cumulative set S0∪ H1∪ H2∪ H3∪ H4, and S0∪ H3 similarly outperformed its multi-step counterpart S0∪ H1∪ H2∪ H3 (Supplementary Table 5).

#### 2.3.4 External wheat dataset enhancement

After four iterations, performance using only GWFSS-derived pseudo-masks plateaued. We therefore introduced an external wheat spike dataset, Global Wheat Head Detection (GWHD) 2021 [14], which provides bounding boxes but no pixel-level masks. Using Model E (fθ4), we generated pseudo-masks on GWHD2021 [14] images:

$$P_{ext}=\{\left(x_{ext},{\hat{y}}_{ext}\right)\;|{\;\hat{y}}_{ext}=f_{\theta T}\left(x_{ext}\right)\text{,}(x_{ext}\in T_{ext}\}$$

P_ext_ denotes the set of pseudo-masks generated on an external dataset GWHD2021. x_ext_ is an image from the GWHD2021 dataset, and y^ext is the pixel-wise pseudo-mask predicted by the current best model f_θT_ for x_ext_.

T_ext_ represents the GWHD2021 image set. After applying the same human screening function Q to P_ext_, we obtained the high-quality external set H_ext_ (another_good_set1), which contains 541 high-quality images. Using these sets, we constructed the final training sets as follows: Model F= S_0_ ∪ H_4_ ∪ H_ext_, Model H (no rice data) = H_4_ ∪ H_ext_, Model G = S_0_ ∪ H_4_ ∪ H_ext_ ∪ S_paddy_, where S_paddy_ is the Paddy rice imagery dataset [3].

#### 2.3.5 Synthetic data experiment (optional)

For comparison, we generated synthetic images by cutting out spike masks from real wheat images (from H_4_ and H_ext_) and pasting them onto five solid-color backgrounds (white, green, gray, yellow, brown). Each original image produced 5 synthetic variants, expanding the dataset 6-fold. Training on this synthetic set gave Model I.

### 2.4 Evaluation metrics

All pseudo-masks were compared against the GWFSS manual annotations (treated as ground truth) using:

Spike IoU = intersection over union for the spike class.

mIoU = mean IoU over spike and background.

Spike Acc = pixel accuracy for spike class.

mAcc = mean pixel accuracy.

### 2.5 Pseudo-mask quality evaluation

We developed a tool called “Mask Quality Screener” to quickly evaluate the quality of spike pseudo-masks. The software UI displays both the original image and the spike mask simultaneously (Fig. 3a). After clicking the “Toggle Mask Overlay” button, the spike mask can be overlaid on the original image (Fig. 3b), facilitating easy comparison of the overlap between the spike mask and the original image. Additionally, clicking the “Flash Mask” button makes the spike mask flash on top of the original image (with adjustable flashing speed) (Supplementary File 1). In this way, evaluating the pseudo-mask quality of a single image takes only 5–10 s.

### 2.6 Benchmarking test

To evaluate the quality of the newly constructed wheat spike pseudo-mask dataset, we performed a benchmark test using two state-of-the-art segmentation models: SegFormer [13] (mit-b0 backbone) and the Swin Transformer (Swin-Tiny [15] with UPerNet). Both models were trained from ImageNet-pretrained weights and evaluated via 3-fold cross-validation, with an 80%/20% train/test split per fold. Training was conducted for 100 epochs with a batch size of 6, an initial learning rate of 6 × 10^−5^, a weight decay of 1 × 10^−4^, and the AdamW optimizer. Input images were resized to 512×512 pixels. Data augmentation included random horizontal flipping, color jitter (brightness and contrast, 0.2), and resizing; no vertical flipping or affine transformations were applied for consistency. The best model per fold was selected based on the validation foreground (spike) IoU, employing an early stopping criterion with a patience of 10 epochs. A cosine annealing learning rate scheduler was used. The loss function was cross-entropy, and outputs were bilinearly upsampled to the original resolution. Evaluation metrics included spike IoU, mean IoU (mIoU), spike pixel accuracy (Spike Acc), and mean accuracy (mAcc). The results (mean ± std over three folds) are reported. This benchmark provides a baseline for comparing the ZCAT-generated pseudo-mask dataset with the manually annotated GWFSS datasets.

Throughout the entire ZCAT pipeline, only the GWFSS original images (without their manual annotations) were used for pseudo-mask generation. The GWFSS manual annotations were strictly withheld and served exclusively as the ground truth for evaluating the pseudo-label datasets. They were never used in model training, pseudo-mask screening, or any other step of the ZCAT pipeline.

We randomly selected 100 images from the FIP 1.0 dataset [16] and performed pixel-level semantic annotation of wheat spikes on them. This curated subset serves as a test set to evaluate the generalization ability of the SegFormer inference models trained on the GWFSS dataset and the ZCAT pseudo-mask datasets (ModelE_pseudo, ModelF_pseudo, ModelG_pseudo, ModelH_pseudo, and ModelI_pseudo). The 100 images from the FIP 1.0 dataset were manually annotated by an expert with 15 years of agronomic and plant science research experience, and the annotations were subsequently verified and corrected by a second expert with 20 years of agronomic background to ensure accuracy. This strictly held-out, expert-labelled wheat spike semantic annotation dataset (comprising 100 images) was maintained completely separate from the ZCAT pipeline throughout the entire process, preventing any data leakage or contamination. Since the SegFormer model was consistently used throughout the ZCAT pipeline for pseudo-mask dataset screening, we adopted the same SegFormer architecture for the generalization tests to maintain methodological consistency.

### 2.7 Time cost assessment between ZCAT and GWFSS manual annotation

We recorded the time consumed during each screening step for the ZCAT subsets (good_set1, good_set2, good_set3, good_set4, and another_goodset1). The cumulative screening time for each stage and the average time cost per pseudo-mask were subsequently calculated (Table 2). Separately, we randomly selected and annotated 50 wheat spike images from the GWFSS dataset, recording the per-image annotation time. The mean value was extrapolated to estimate the total time required for fully annotating the entire 739-image GWFSS dataset. These measurements enabled a direct baseline comparison of total and per-image time costs between the ZCAT screening pipeline and manual GWFSS annotation, as presented in Supplementary Fig. 1.

**Table 2:** Accuracy of pseudo-masks relative to GWFSS ground truth for different training data combinations. The total time recorded from ModelA_pseudo through ModelI_pseudo represents the cumulative effort required to generate the corresponding pseudo-masks. For each pseudo-mask stage, the per-image time is obtained by dividing the total screening time by the number of images processed up to that stage.

Metric	Model A	Model B	Model C	Model D	Model E	Model F	Model G	Model H	Model I
Spike IoU	0.4785	0.6576	0.6725	0.6939	0.7003	0.7046	0.7025	0.7048	0.6996
mIoU	0.6929	0.7954	0.8038	0.8189	0.8234	0.8264	0.8244	0.826	0.8234
Spike Acc	0.5801	0.7856	0.7976	0.8015	0.7881	0.7878	0.7947	0.7908	0.781
mAcc	0.7697	0.8731	0.8791	0.8859	0.8815	0.882	0.8844	0.8842	0.8803
Model trained from	CVRP + RiceSEG	CVRP + RiceSEG + good_set1	CVRP + RiceSEG + good_set2	CVRP + RiceSEG + good_set3	CVRP + RiceSEG + good_set4	CVRP + RiceSEG + good_set4+another_goodset1	CVRP + RiceSEG + good_set4+another_goodset1+Paddy rice imagery dataset	good_set4+another_good_set1	The synthetic dataset of good_set4+another_good_set1
Predicted Mask	ModelA_pseudo	ModelB_pseudo	ModelC_pseudo	ModelD_pseudo	ModelE_pseudo	ModelF_pseudo	ModelG_pseudo	ModelH_pseudo	ModelI_pseudo
Total time consumed to obtain pseudo-labels (s)	0	4482	9588	14,680	20,354	29,950	29,950	29,950	29,950
Time per image (s)	0	6.0649	6.4871	6.6215	6.8856	7.1242	7.1242	7.1242	7.1242

### 2.8 UMAP visualization

To qualitatively assess the diversity introduced by the external GWHD2021 dataset, a UMAP projection was performed on the original RGB images of another_goodset1 (541 images) and the GWFSS dataset (739 images). Image features were extracted using a ResNet-50 backbone pre-trained on ImageNet (ResNet50_Weights. IMAGENET1K_V1) with the final fully connected layer removed, yielding 2048-dimensional feature vectors via global average pooling. All images were resized to 224×224 pixels and normalized with ImageNet statistics (mean = [0.485, 0.456, 0.406], std = [0.229, 0.224, 0.225]). The feature vectors were standardized to zero mean and unit variance using StandardScaler, and then reduced to two dimensions with UMAP using the following parameters: n_components = 2, random_state = 42, n_neighbors = 15, min_dist = 0.1, metric = 'euclidean’, and init = 'spectral’. The resulting embedding was visualized with scatter plots, comparing the coverage of the two datasets.

## 3 Results

### 3.1 Zero-annotation generation of wheat spike pseudo-masks

We first evaluated the three rice panicle datasets. CVRP and RiceSEG have high resolution and good annotation quality; the Paddy rice dataset has blurry images and incomplete annotations (Fig. 1).

**Fig. 4:**
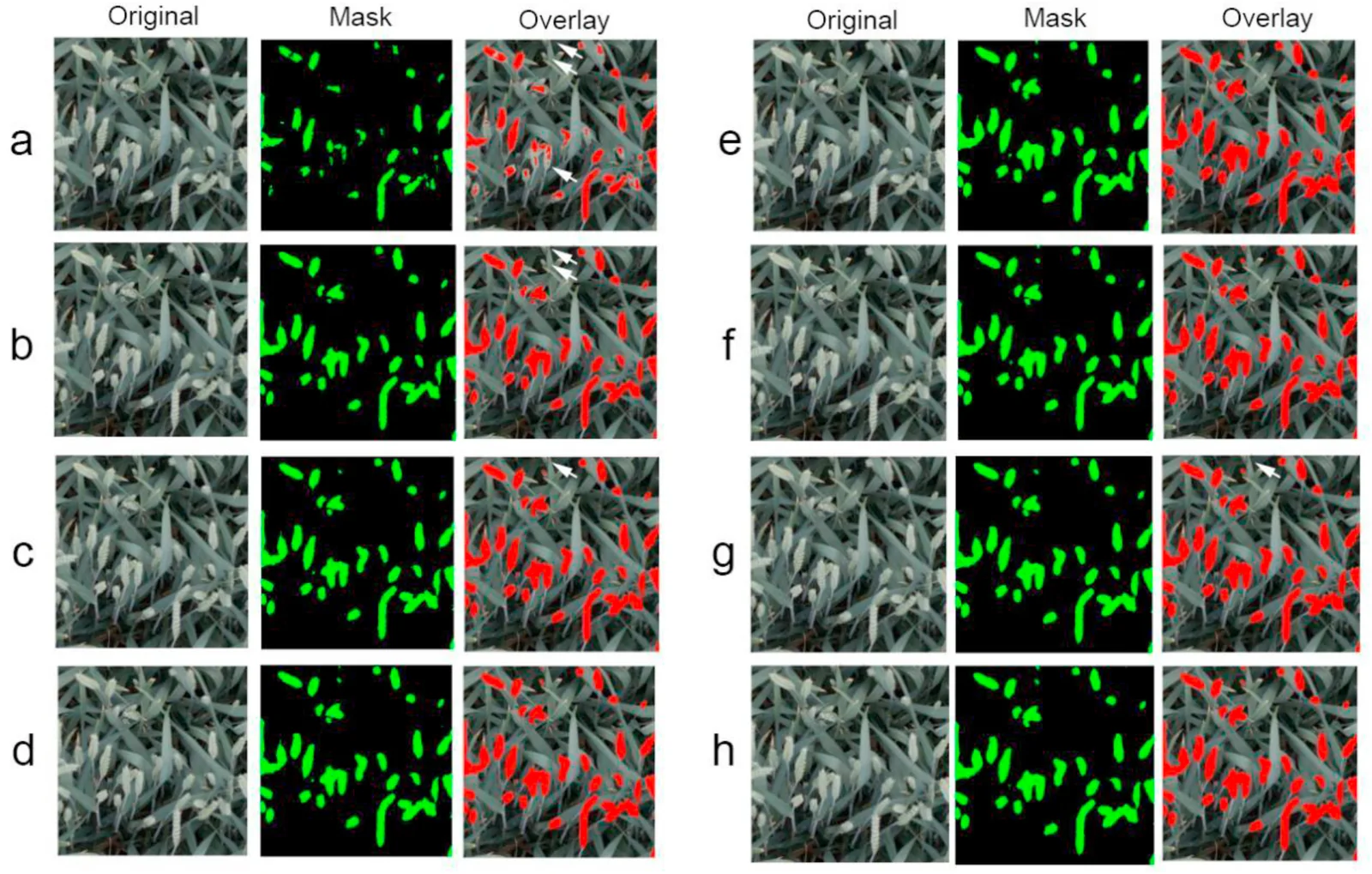
Pseudo masks generated by models A to H through inference on the GWFSS dataset. For each model, the left, middle, and right columns show the original image, the pseudo mask, and their overlay, respectively. The same wheat image is used to demonstrate the spike recognition capability of models A to H. (a) Inference result of model A trained on the CVRP and RiceSEG datasets; (b) model B trained on CVRP + RiceSEG + good_set1; (c) model C trained on CVRP + RiceSEG + good_set2; (d) model D trained on CVRP + RiceSEG + good_set3; (e) model E trained on CVRP + RiceSEG + good_set4; (f) model F trained on CVRP + RiceSEG + good_set4 + another_good_set1; (g) model G trained on CVRP + RiceSEG + good_set4 + another_good_set1 + Paddy rice imagery (five datasets in total); (h) model H trained on good_set4 + another_good_set1. CVRP and RiceSEG are two open-source rice panicle semantic segmentation datasets, and GWFSS is a wheat spike semantic segmentation dataset. Good_set1 to good_set4 are high-quality pseudo-mask datasets selected from the pseudo masks inferred by models A to D on GWFSS, containing 157, 179, 242, and 230 images, respectively. Another_good_set1 consists of 541 high-quality images screened from the pseudo masks generated by model E (trained on CVRP + RiceSEG + good_set4) when inferring spikes on the GWHD2021 dataset.

To preliminarily verify the feasibility of cross-crop transfer, we merged the two rice datasets (CVRP + RiceSEG, total 2857 images) and trained a SegFormer-based panicle segmentation model, denoted as Model A. Model A was then applied to the GWFSS wheat dataset to generate spike pseudo-masks, forming pseudo-mask dataset A. Comparing these pseudo-masks with the GWFSS manual annotations yielded a Spike IoU of 0.4785 and an mIoU of 0.6929 (Table 2), highlighting a substantial cross-crop transfer challenge when directly applying a rice panicle model to wheat. This challenge is visually illustrated in Fig. 4, which compares different models on a common image. Specifically, as shown in Fig. 4a, Model A's inference performance is mediocre, as indicated by the areas pointed to by multiple arrows.

To improve performance on the target domain (wheat), we manually screened 157 good-quality pseudo-mask images from dataset A to construct good_set1. Merging good_set1 with the original rice datasets (CVRP + RiceSEG) and retraining produced Model B. Model B generated pseudo-mask dataset B on GWFSS, which achieved a Spike IoU of 0.6576 and mIoU of 0.7954. This shows that only a small number (157) of manually screened high-quality pseudo-masks (without any pixel-level annotation) can significantly enhance model generalization in the target domain, validating the effectiveness of human-guided refinement: a small amount of reliable pseudo-masks are more valuable than a large number of noisy labels. From Fig. 4b, it can be seen that the inference performance of model B is significantly better than that of model A, but there are still two areas indicated by arrows where the spikes are not recognized.

Following the same iterative strategy, we successively added the subsequent high-quality pseudo-masks (good_set2, good_set3, good_set4) to the training set, retraining Models C, D, and E. The performance of the pseudo-masks generated by these models on GWFSS is shown in Table 2: Spike IoU improved from 0.6725 (Model C) to 0.7003 (Model E), and mIoU from 0.8038 to 0.8234. This trend confirms that manual screening of high-quality pseudo-masks steadily enhances spike recognition. From Fig. 4c, it can be seen that the inference performance of model C is even better than that of model B, with only one area indicated by an arrow where the spike is not recognized. From Fig. 4d to e, the recognition performance of models D and E is already comparable to manual annotation.

However, after four iterations, further improvements from screening only within GWFSS became marginal. We therefore introduced the GWHD2021 dataset [14]. Using Model E (trained on CVRP + RiceSEG + good_set4), we performed pseudo-mask inference on GWHD2021 images and selected 541 high-quality pseudo-masks, denoted as another_good_set1. Merging another_good_set1 with the previous training set (CVRP + RiceSEG + good_set4) and retraining gave Model F. Model F's pseudo-masks on GWFSS showed further improvement: Spike IoU reached 0.7046 and mIoU 0.8264. This indicates that incorporating external wheat pseudo-masks is more effective than relying solely on GWFSS internal screening. Fig. 4e to f shows that the inference performance of models E and F is comparable.

We further investigated whether adding more rice panicle data would still benefit wheat panicle recognition. Adding the Paddy rice imagery dataset to the training set (CVRP + RiceSEG + good_set4+another_good_set1) and training Model G gave a Spike IoU of 0.7025 on GWFSS, slightly lower than Model F's 0.7046. This is likely due to the lower quality of the Paddy rice dataset (Fig. 1c), and also suggests that once sufficient wheat pseudo-masks are available, additional rice data may no longer bring gains. Fig. 4f and g shows that the inference performance of model G declines compared to model F, as seen in the areas indicated by arrows in Fig. 4g.

We also compared Model F (which included rice data) with Model H (trained only on good_set4+another_good_set1, without any rice data). Model H achieved a Spike IoU of 0.7048, slightly higher than Model F's 0.7046, indicating that when enough wheat pseudo-masks are available, removing rice data is beneficial for wheat spike segmentation accuracy. Fig. 4f and h show that the inference performance of model H is comparable to that of model F.

**Fig. 5:**
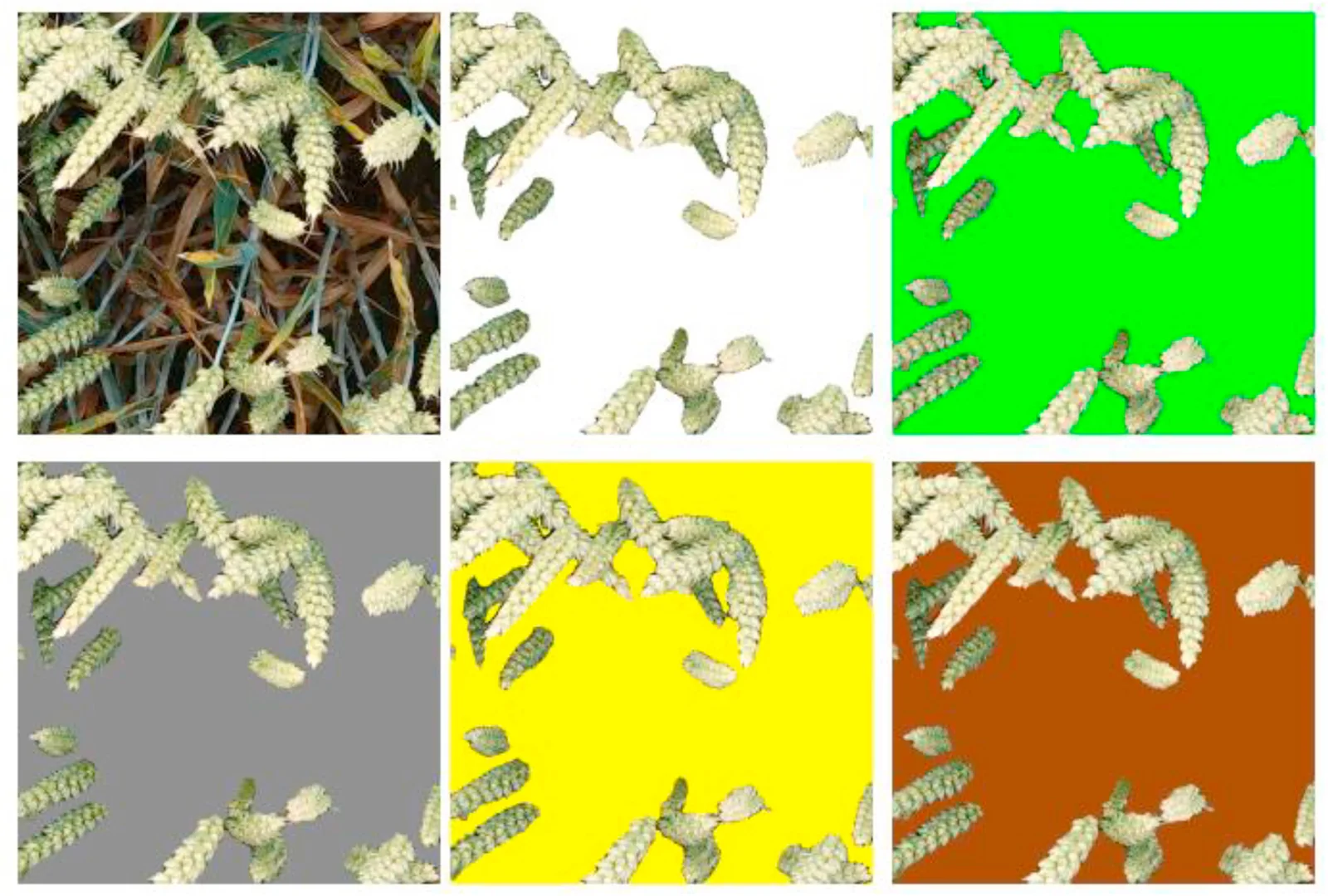
Image augmentation method by simple background replacement.

**Fig. 6:**
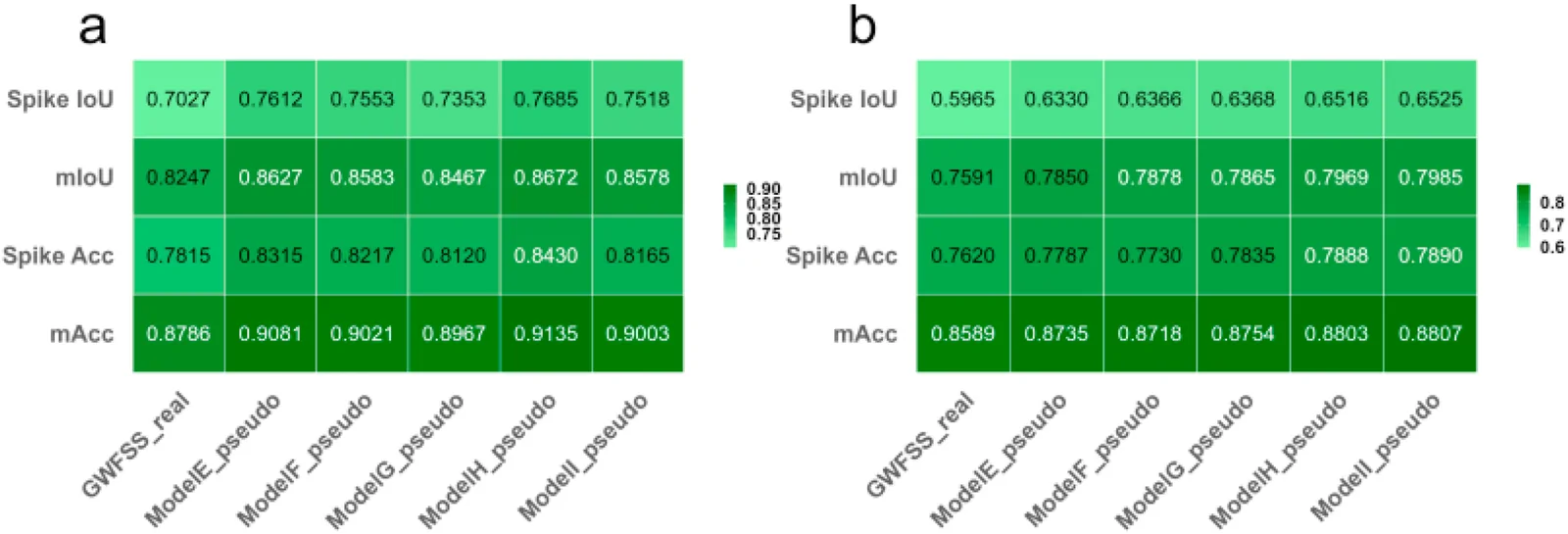
Benchmark performance of SegFormer (a) and Swin-Transformer (b) on different datasets including GWFSS_real, ModelE_pseudo, ModelF_pseudo, ModelG_pseudo, ModelH_pseudo, and ModelI_pseudo. GWFSS_real is the human-labelled spike semantic segmentation dataset. ModelE_pseudo to ModelI_pseudo are pseudo-masks inferred by applying the corresponding model to the GWFSS images. See Table 2 for the datasets used to train models E to I.

Finally, we tested synthetic data augmentation. Using the real wheat images and masks from good_set4+another_good_set1, we replaced the background with five solid colors (Fig. 5), expanding the 541 images to 3246 images. Training Model I on this synthetic dataset yielded a Spike IoU of 0.6996 on GWFSS, lower than Model H's 0.7048, showing that simple background replacement did not improve wheat panicle recognition. Notably, although this synthetic augmentation did not improve the recognition ability of Model I itself, the pseudo-mask dataset generated by these synthetic data (ModelI_pseudo) still exhibited slightly higher benchmark performance than the manually annotated GWFSS ground truth (Spike IoU 0.7518 vs. 0.7027, Fig. 6).

### 3.2 Benchmark and generalization performance of ZCAT-generated pseudo-masks against GWFSS manual annotations

We benchmarked several pseudo-mask datasets (ModelE_pseudo through ModelI_pseudo) using SegFormer and Swin-Transformer, comparing them against the GWFSS manual annotations (GWFSS_real). As shown in Fig. 6, all pseudo-mask datasets had higher baseline than the manual annotations in Spike IoU, mIoU, Spike Acc, and mAcc. This demonstrates that zero-annotation wheat spike pseudo-masks, generated via cross-crop transfer, show higher self-consistency than the manual annotations. We randomly selected 100 images from the FIP-1.0 dataset and manually annotated the wheat spikes. This curated subset served strictly as a held-out test set to evaluate the generalization ability of the SegFormer models trained on the GWFSS manual annotations and the ZCAT pseudo-mask datasets (ModelE_pseudo to ModelI_pseudo). As a result, all ZCAT pseudo-mask datasets achieved higher Spike IoU and mIoU than the GWFSS manual annotations on this test set.

**Fig. 7:**
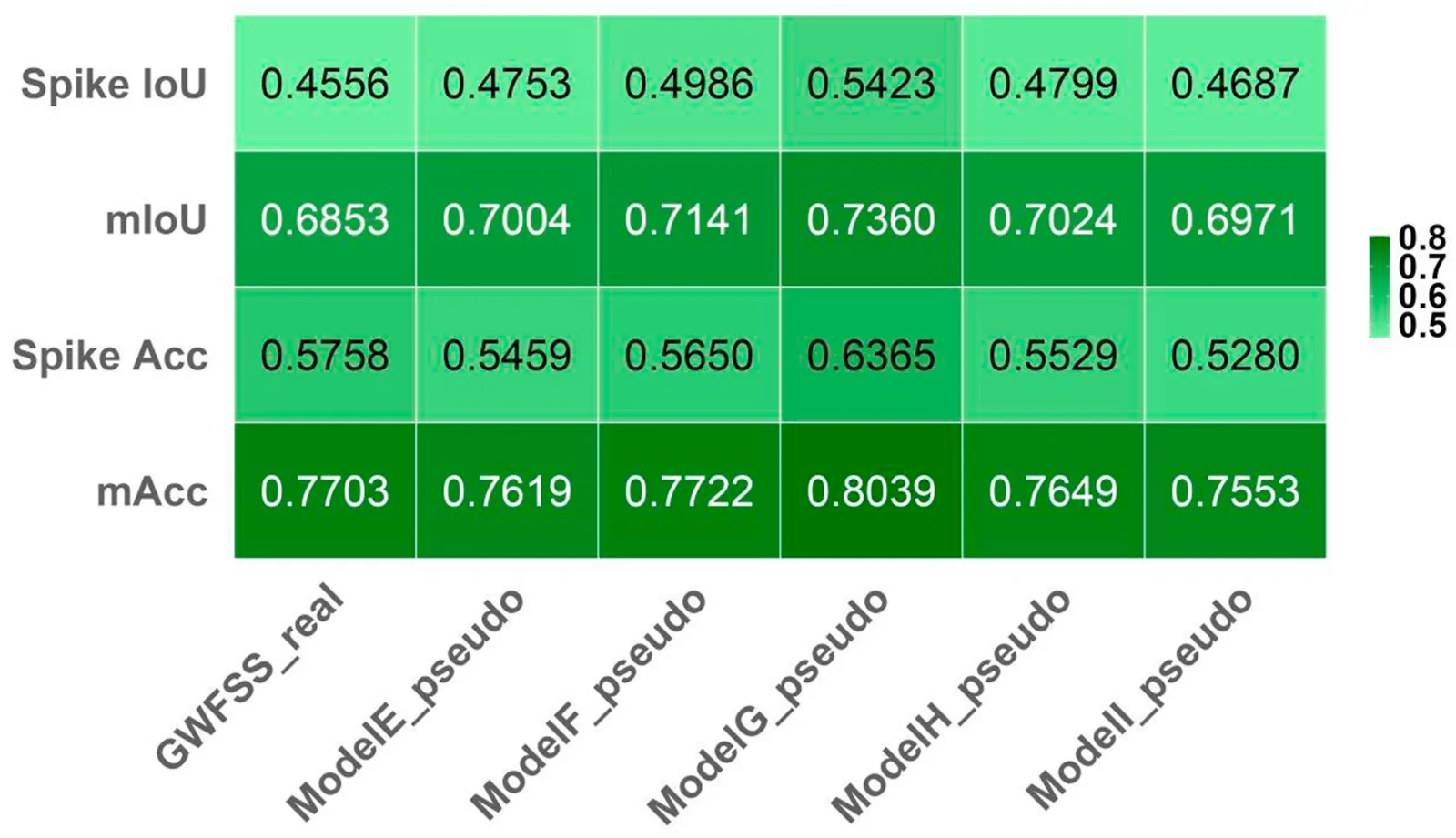
Generalization ability test of the GWFSS_real (human-labelled) and five ZCAT pseudo-mask datasets (ModelE_pseudo, ModelF_pseudo, ModelG_pseudo, ModelH_pseudo, and ModelI_pseudo) by SegFormer. A strictly held-out, expert-labelled FIP-1.0 dataset was used as the test set.

To compare time costs, we recorded screening time for each ZCAT stage (good_set1 to good_set4 and another_goodset1) and derived the average time per pseudo-mask (Table 2). We also annotated 50 GWFSS images manually, averaged the per-image time, and projected this to all 739 images. The total manual annotation time was 229,090 s, versus 20,354 s for ModelE_pseudo (only 8.89% of the manual effort), while achieving comparable benchmark and generalization performance (Supplementary Fig. 1, Figs. 6, Fig.7). Average screening time per pseudo-mask was about 6–7 s, compared with ∼310 s per manual annotation (Supplementary Fig. 1).

### 3.3 High-quality wheat spike pseudo-mask dataset

Through the iterative screening process described above, we selected 541 high-quality spike images from the GWHD2021 pseudo-masks to form the final wheat spike pixel-wise pseudo-mask dataset (another_good_set1). Benchmarking this dataset with SegFormer (Table 3) gave a Spike IoU of 0.7753 ± 0.0107, mIoU of 0.8712 ± 0.0053, Spike Acc of 0.8139 ± 0.0251, and mAcc of 0.9030 ± 0.0108. All images in another_good_set1 dataset were manually verified to ensure the quality of the pseudo-masks.

**Table 3:** Benchmark performance (mean ± std) of the final generated wheat spike pseudo-mask dataset (another_good_set1).

Metric	Segformer	Swin-Transformer
Spike IoU	0.7753 ± 0.0107	0.7748 ± 0.0118
mIoU	0.8712 ± 0.0053	0.8682 ± 0.0067
Spike Acc	0.8139 ± 0.0251	0.8794 ± 0.0102
mAcc	0.9030 ± 0.0108	0.9296 ± 0.0050

**Fig. 8:**
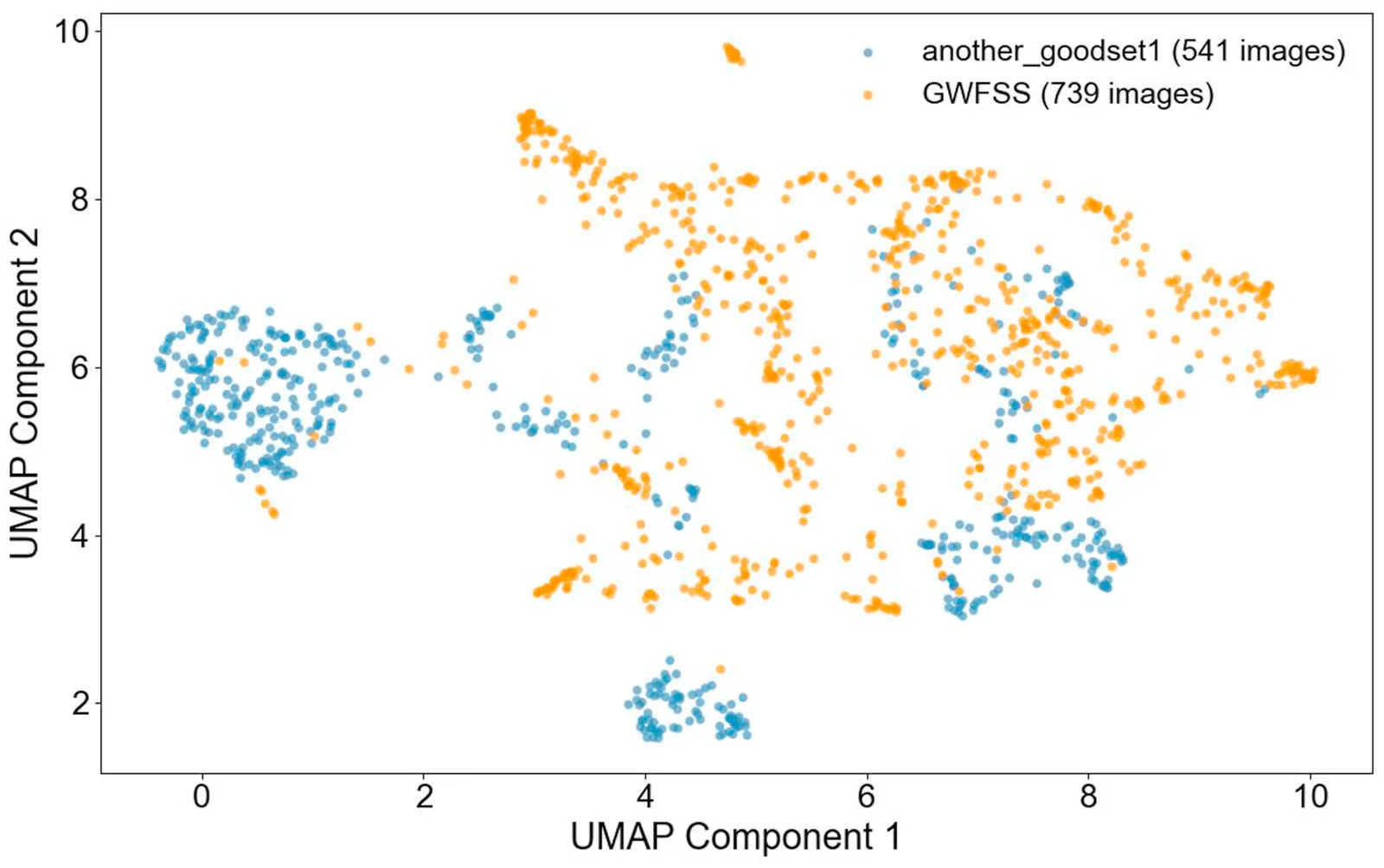
UMAP visualization of the original images from GWFSS dataset (yellow) and another_good_set1 (blue). Features were extracted with a pre-trained ResNet-50 and reduced using UMAP (n_neighbors = 15, min_dist = 0.1, Euclidean metric, spectral initialization). The blue points extend into some regions not covered by the yellow points, indicating that the inclusion of external GWHD2021 (source of another_good_set1) data introduces additional image diversity beyond the original GWFSS distribution.

To further demonstrate the diversity contributed by the external dataset, we performed UMAP visualization on the original images of another_good_set1 (blue) and GWFSS (yellow). As shown in Fig. 8, the blue points of another_good_set1 extend into regions not occupied by the yellow points. This indicates that by incorporating the external GWHD2021 dataset, another_good_set1 successfully covers a broader range of image types, thereby providing a richer data foundation for subsequent cross-crop transfer learning.

## 4 Discussion

### 4.1 Effectiveness of human-guided iterative refinement

Our results demonstrate that iterative pseudo-mask screening with human quality assessment successfully transfers a rice panicle segmentation model to wheat spikes, generating a pseudo-mask dataset that achieves performance comparable with, and in our specific setting slightly better than, manual annotations. This aligns with Cao et al. [11] that “label quality sometimes outweighs label quantity”. Using only 157 high-quality pseudo-masks (good_set1) improved Spike IoU from 0.4785 to 0.6576; a small number of reliable pseudo-masks provides effective “anchors” for domain adaptation.

Our method achieves three breakthroughs: (1) Complete elimination of target domain pixel-level annotation. Human effort reduces from pixel-level drawing to holistic quality assessment (seconds per image). (2) Cross-crop knowledge transfer. We leverage morphological similarity between rice panicles and wheat spikes, transferring annotations across species for the first time. (3) Iterative refinement with human guidance. Multi-round screening continuously improves performance (Table 2).

It is important to note that our cross-validation benchmarking (Fig. 6 and Table 3) employed a standard random 3-fold split without explicitly stratifying by experimental site or image acquisition conditions. Consequently, this may introduce a potential site-level data leakage, which could lead to a slight overestimation of the absolute metric values. However, because the GWFSS manual annotations and all five ZCAT-generated pseudo-mask datasets (ModelE_pseudo to ModelI_pseudo) share exactly the same original images and undergo the identical cross-validation fold assignments, any leakage-induced bias is systematically applied across all evaluated datasets. Therefore, the relative superiority of the ZCAT pseudo-masks over the manual annotations, demonstrated by consistently higher Spike IoU and mIoU, remains robust and unaffected. Moreover, a strictly held-out FIP-1.0 test set (100 images, completely isolated from the training pipeline) provided a leakage-free evaluation, where the ZCAT-trained model still performed slightly better than the manually annotated counterpart, further confirming the potential of ZCAT.

We attribute the success of ZCAT to several key factors. First, despite the morphological differences between rice panicles and wheat spikes, the two organs share common structural characteristics (e.g., elongated branching structures and compact spatial arrangements). Second, the SegFormer architecture, with its self-attention mechanism, effectively captures global contextual information and long-range dependencies, facilitating the recognition of these cross-crop shape similarities. Third, we were encouraged to find that the initial model, trained solely on rice data, already produced a substantial number of reasonably good pseudo-masks on wheat (157 out of 739 images), providing a sufficient “seed” set to initiate iterative refinement. Fourth, the human-guided screening process, combined with the non-accumulating curriculum design, ensured that only high-quality pseudo-masks were used for retraining in each round, thereby preventing error accumulation and enabling continuous performance improvement.

### 4.2 Insights from synthetic data

Although simple background replacement did not improve model performance (Model I Spike IoU 0.6996 vs. Model H 0.7048), the pseudo-masks dataset generated by Model I exhibited higher benchmark scores than the manual annotated GWFSS ground truth (0.7518 vs. 0.7027). This apparent paradox can be explained by distinguishing between model inference performance and dataset quality as training data.

We attribute this to several possible reasons. First, the synthetic images were generated by replacing the original backgrounds with solid colors, effectively performing “de-backgrounding”. This process reduced the availability of background cues, forcing the model to focus more on the intrinsic shape features of spikes rather than on context. Consequently, the model became less prone to overfitting specific backgrounds, resulting in a pseudo-mask dataset with more consistent and less background-biased predictions. Second, the synthetic augmentation introduced systematic error patterns (e.g., unnaturally sharp boundaries and occasional mis-segmentation of background regions). Because these errors were consistent across all synthetic samples, they functioned as a form of regularization; the model learned to disregard these recurrent artifacts and instead extracted more robust, generalizable spike representations. This prevented the model from memorizing training-specific patterns and improved its ability to generalize to unseen images. Third, the increased sample size (from 541 to 3246 images) compensated for the slightly lower per-sample quality, as benchmark performance ultimately depends on both label quality and dataset diversity. In other words, a large-scale dataset with slightly lower but consistent pseudo-label quality can outperform a smaller dataset with higher per-sample accuracy when used for training a segmentation model.

### 4.3 Comparison with existing methods

Table 4 summarizes the key differences between our ZCAT paradigm and six representative manual or semi-automatic annotation approaches, from which ZCAT exhibits three distinct advantages. First, it completely eliminates target-domain pixel-level annotation. Second, ZCAT enables cross-crop transferability. Third, ZCAT achieves iterative refinement that yields performance competitive with or slightly better than manual annotations in our specific setting. Beyond these five methods, ZCAT also offers advantages over other weakly supervised or generative approaches: unlike unsupervised domain adaptation using GANs [7] (which require additional generators and may distort organ structure), ZCAT directly adapts the model through pseudo-mask screening and human-guided retraining, avoiding semantic distortion; unlike structure-generation methods like LeafGen [10] (which require predefined L-system rules and cannot transfer across species), ZCAT directly reuses existing annotated datasets from one crop to generate annotations for another, with lower computational cost and simpler workflow. Our ZCAT paradigm also differs fundamentally from recently proposed depth-based pseudo-labeling methods such as DepthCropSeg++ [17]. DepthCropSeg++ generates pseudo-masks from monocular depth maps using Depth Anything V2, followed by a two-stage self-training strategy that refines labels via consistency checking. This approach requires reliable depth estimation and careful post-processing to handle scenes with dense canopies or reflective surfaces. In contrast, ZCAT circumvents depth estimation altogether by leveraging the morphological similarity between rice panicles and wheat spikes, and uses a simple binary quality function Q (good/bad) to filter pseudo-masks through human visual inspection. While DepthCropSeg++ excels in scaling to a large number of species and environments (28,406 images, 30+ species), ZCAT offers a lightweight alternative that requires no target-domain pixel-level labels and incurs minimal human effort (5-10 s per image for quality assessment). The two approaches are therefore complementary: DepthCropSeg++ is well-suited for building general-purpose crop segmentation foundation models, whereas ZCAT is specifically designed for rapid, low-cost generation of organ-level datasets for specific target crops.

**Table 4:** Comparison of the proposed ZCAT paradigm with other similar manual or semi-automatic annotation paradigms.

Aspect	RiceSEG [1]	GWFSS [4]	CVRP [2]	Paddy rice dataset [3]	Gao et al. (self-supervised) [11]	DepthCropSeg++ [17]	ZCAT paradigm
Need manual pixel annotation?	Yes	Yes	Yes	Yes	No	No	No
Cross-crop transfer?	No	No	No	No	No	Yes	Yes
Iterative mechanism	None	None	None	None	Yes	Yes	Yes
Final output performance	Spike IoU = 0.7027	SegFormer leaf/spike IoU>80%	Near manual	Semi-auto > manual (precision)	Near manual	93.11% mIoU	Comparable to or better than manual (in our setting)

### 4.4 Transformer architecture

SegFormer's self-attention mechanism captures global context, facilitating recognition of shape similarities between rice panicles and wheat spikes, consistent with findings in RiceSEG [1] and CVRP [2] that Transformers outperform CNNs on ear segmentation. The key to SegFormer's success in this work, however, is the introduction of a human-defined quality function Q, which solves a fundamental self-learning bottleneck: without ground-truth labels, computers cannot autonomously distinguish correct pseudo-masks from incorrect ones. Conventional pipelines retrain on all predictions, causing error accumulation, whereas ZCAT selects only high-quality pseudo-masks through human visual inspection, transforming iterative refinement into a curriculum learning strategy that continuously improves performance. Q thus provides an oracle signal that computers cannot generate on their own, as evaluating pseudo-mask quality requires semantic understanding of shape, boundary, and occlusion, subtle cues that humans naturally integrate but machines cannot judge without reference labels. This human-in-the-loop solution is therefore central to the success of ZCAT.

### 4.5 Limitations and future work

From our practical implementation, we have identified several limitations of the current ZCAT framework. First, the quality of the final pseudo-mask dataset is closely tied to the screening criteria defined by human experts (the Q function), with stricter criteria yielding higher-quality pseudo-masks, whereas looser criteria may introduce more noise. Second, the characteristics of the resulting pseudo-mask dataset are influenced by the selection bias of individual experts, as an expert who tolerates more false negatives (FN) will produce a dataset with systematically under-segmented masks. Third, despite multiple iterations, the SegFormer model trained on ZCAT-generated pseudo-masks still struggles with certain challenging wheat spike images, particularly those with senescent spikes against near-uniform backgrounds, and these images were consistently excluded from the goodset across all iterations, indicating that ZCAT alone may not suffice for these hard cases. Fourth, due to the scarcity of open-source organ-level segmentation datasets for other crops, we have only demonstrated ZCAT on rice-to-wheat transfer, and its applicability to other crop-organ pairs (e.g., rice to sorghum, maize, or other cereal crops) remains unclear.

Future work could also: (1) use more advanced foundation models, as suggested by Ubbens et al. [18]; (2) incorporate more diverse wheat varieties and growth stages; (3) develop more efficient screening strategies; (4) combine pseudo-masks with small amounts of real annotations (5) explore multi-view imaging [19] and 3D reconstruction techniques (e.g., NeRF, 3DGS [20]); to synthesize complete spike geometries from limited viewpoints, thereby generating high-quality pseudo-masks without manual annotation, as occluded or partially visible spikes can be reconstructed from complementary views.

### 4.6 Data openness

An open data culture is crucial for accelerating the development of deep learning algorithms in plant phenotyping research [11,21,22]. This study would not have been possible without open-source datasets such as CVRP [2], RiceSEG [1], Paddy rice imagery dataset [3], and GWFSS [4]. Therefore, we have open-sourced the wheat spike pixel-wise pseudo-mask dataset (541 images) generated in this study, as well as the Mask Quality Screener tool, to support further research on cross-crop organ segmentation and zero-annotation learning. The Mask Quality Screener is released under the MIT License to facilitate broad adoption and further development by the research community. We call upon researchers in plant phenotyping and digital agriculture to foster an open-sharing culture, thereby advancing precision agriculture and crop breeding technologies.

## 5 Conclusion

We proposed ZCAT, a zero-annotation cross-crop pseudo-mask screening paradigm that replaces pixel-level annotation with simple mask quality assessment. By introducing a human-defined quality function Q, ZCAT circumvents the key challenge in self-learning: the inability of computers to autonomously distinguish good masks from bad ones. Training an initial model on open-source rice panicle datasets, combined with human holistic quality assessment and curriculum learning-based iterative refinement, we generated a wheat spike segmentation dataset that shows competitive or slightly better performance than manual annotations in our benchmarks (Spike IoU 0.7753). We further show that incorporating additional datasets expands the diversity of pseudo-masks. In short, ZCAT (1) produces pseudo-masks that achieve benchmark performance comparable to or slightly better than manual annotations without any target-domain labels, solely by leveraging open-source data of morphologically similar organs; and (2) enriches the pseudo-mask collection using extra datasets, ultimately building a large-scale, diverse, and high-quality pseudo-mask repository. ZCAT completely eliminates pixel-level manual annotation on the target crop, reducing annotation cost from hours to seconds, opens a new pathway for rapid plant organ annotation, and is expected to significantly lower data preparation costs in plant phenomics research.

## Author contributions

Y.Z, and G.F. wrote the paper. T. C. and W. F. crafted all figures and tables. Y. Z., G. F., and S. C., designed the whole architecture of the proposed methodology. Y. Z. and T. W. carried out all the experiments. Y. Z. prepared the dataset. Y. Z. and G. F. organized the references. G. F. and T. C. provided funding support. All authors read and approved the final manuscript.

## Data availability

The wheat spike pseudo-mask dataset and Mask Quality Screener are available at https://github.com/zyxyes1/MaskQualityScreener and https://github.com/zyxyes1/Wheat-Spike-Semantic-Segmentation, respectively.

## Declaration of competing interests

The authors declare that they have no known competing financial interests or personal relationships that could have appeared to influence the work reported in this paper.
