# Supplementary material for: ZCAT: Zero-shot cross-crop annotation transfer–A new paradigm leveraging plant organ similarity
Source: Plant Phenomics. 2026 Aug 13;8(4):100267. doi: 10.1016/j.plaphe.2026.100267 (PMC13521136; doi:10.1016/j.plaphe.2026.100267)
Supplement: Supplementary file 1 [file mmc2.docx]

**Supplementary Table 1**. Comparison between good_set1 and bad_set1 with the corresponding ground-truth masks using Welch's t-test.

| Metric | Good_set1 | Bad_set1 | P value |
| --- | --- | --- | --- |
| Spike IoU | 0.7967 | 0.3927 | <0.01 |
| mIoU | 0.8744 | 0.6439 | <0.01 |
| Spike Acc | 0.859 | 0.5048 | <0.01 |
| mAcc | 0.9192 | 0.7293 | <0.01 |

**Supplementary Table 2**. Comparison between good_set2 and bad_set2 with the corresponding ground-truth masks using Welch's t-test.

| Metric | Good_set2 | Bad_set2 | P value |
| --- | --- | --- | --- |
| Spike IoU | 0.8088 | 0.6093 | <0.01 |
| mIoU | 0.8875 | 0.766 | <0.01 |
| Spike Acc | 0.8393 | 0.7684 | <0.01 |
| mAcc | 0.9154 | 0.8596 | <0.01 |

**Supplementary Table 3**. Comparison between good_set3 and bad_set3 with the corresponding ground-truth masks using Welch's t-test.

| Metric | Good_set3 | Bad_set3 | P value |
| --- | --- | --- | --- |
| Spike IoU | 0.8073 | 0.6067 | <0.01 |
| mIoU | 0.8869 | 0.7633 | <0.01 |
| Spike Acc | 0.8468 | 0.7735 | <0.01 |
| mAcc | 0.9181 | 0.8602 | <0.01 |

**Supplementary Table 4**. Comparison between good_set4 and bad_set4 with the corresponding ground-truth masks using Welch's t-test.

| Metric | Good_set4 | Bad_set4 | P value |
| --- | --- | --- | --- |
| Spike IoU | 0.8122 | 0.6405 | <0.01 |
| mIoU | 0.8897 | 0.7869 | <0.01 |
| Spike Acc | 0.8565 | 0.7766 | <0.01 |
| mAcc | 0.9226 | 0.8693 | <0.01 |

**Supplementary Table 5**. Comparison of spike pseudo-masks generated on GWFSS with the corresponding ground-truth masks, for models trained with different data combinations. For instance, the model trained on “CVRP+RiceSEG+good_set1” was used to infer pseudo-masks for GWFSS images; the reported metrics (Spike IoU, mIoU, Spike Acc, and mAcc) are averages computed over all images.

| Model trained from | CVRP+RiceSEG+good_set1 | CVRP+RiceSEG+good_set1+good_set2 | CVRP+RiceSEG+good_set2 | CVRP+RiceSEG+good_set1+good_set2+good_set3 | CVRP+RiceSEG+good_set3 | CVRP+RiceSEG+good_set1+good_set2+good_set3+good_set4 | CVRP+RiceSEG+good_set4 |
| --- | --- | --- | --- | --- | --- | --- | --- |
| Spike IoU | 0.6576 | 0.6811 | 0.6725 | 0.6889 | 0.6939 | 0.6945 | 0.7003 |
| mIoU | 0.7954 | 0.8091 | 0.8038 | 0.8164 | 0.8189 | 0.8199 | 0.8234 |
| Spike Acc | 0.7856 | 0.8050 | 0.7976 | 0.7822 | 0.8015 | 0.7857 | 0.7881 |
| mAcc | 0.8731 | 0.8838 | 0.8791 | 0.8772 | 0.8859 | 0.8794 | 0.8815 |

CVRP and RiceSEG are two datasets with manually annotated rice panicles. The four sets, good_set1 through good_set4, are high-quality pseudo-masks selected from the pseudo-masks inferred on GWFSS by Segformer models trained on four different training combinations, namely: “CVRP+RiceSEG”, “CVRP+RiceSEG+good_set1”, “CVRP+RiceSEG+good_set2”, and “CVRP+RiceSEG+good_set3”, respectively.

**Supplementary Figure 1**. Comparison of total and per-image human effort between manual annotation and iterative pseudo-mask screening. The cumulative time for each pseudo-mask stage (ModelA_pseudo to ModelI_pseudo) represents the total screening effort invested up to that stage. Per-image time for each stage is calculated as the cumulative screening time divided by the number of images processed at that stage. The constant total time for Models F through I reflects that no additional human screening was conducted after Model F; hence, the cumulative effort for subsequent pseudo-mask sets remains unchanged. To estimate the total annotation time for the full GWFSS dataset (739 images), we randomly selected 50 wheat spike images, measured the average annotation time per image, and extrapolated this value to the entire dataset, yielding both the total and per-image time costs for the GWFSS manual annotations. All values are reported in seconds.
